# Isolation and preliminary characterization of a novel bacteriophage vB_KquU_φKuK6 that infects the multidrug-resistant pathogen *Klebsiella quasipneumoniae*

**DOI:** 10.3389/fmicb.2024.1472729

**Published:** 2024-10-15

**Authors:** Isaac P. Miller, Alma G. Laney, Geoffrey Zahn, Brock J. Sheehan, Kiara V. Whitley, Ruhul H. Kuddus

**Affiliations:** Department of Biology, Utah Valley University, Orem, UT, United States

**Keywords:** *Klebsiella quasipneumoniae*, MDR bacterial pathogens, phage therapy, lytic/lysogenic infection cycle, biofilm, phage-antibiotic synergy

## Abstract

**Background:**

*Klebsiella quasipneumoniae* (previously known as *K. pneumoniae* K6) strains are among the multidrug-resistant hypervirulent bacterial pathogens. Phage therapy can help treat infections caused by such pathogens. Here we report some aspects of virology and therapeutic potentials of vB_KquU_φKuK6, a bacteriophage that infects *Klebsiella quasipneumoniae.*

**Methods:**

*K. quasipneumoniae* (ATCC 700603) was used to screen wastewater lytic phages. The isolate vB_KquU_φKuK6 that consistently created large clear plaques was characterized using standard virological and molecular methods.

**Results:**

vB_KquU_φKuK6 has a complex capsid with an icosahedral head (~60 nm) and a slender tail (~140 nm × 10 nm). The phage has a 51% AT-rich linear dsDNA genome (51,251 bp) containing 121 open reading frames. The genome contains genes encoding spanin, endolysin, and holin proteins necessary for lytic infection and a recombinase gene possibly involved in lysogenic infection. vB_KquU_φKuK6 is stable at −80 to +67°C, pH 4–9, and brief exposure to one volume percent of chloroform. vB_KquU_φKuK6 has a narrow host range. Its lytic infection cycle involves a latency of 20 min and a burst size of 435 plaque-forming units. The phage can cause lysogenic infection, and the resulting lysogens are resistant to lytic infection by vB_KquU_φKuK6. vB_KquU_φKuK6 reduces the host cells’ ability to form biofilm but fails to eliminate that ability. vB_KquU_φKuK6 demonstrates phage-antibiotic synergy and reduces the minimum inhibitory concentration of chloramphenicol and neomycin sulfate by about 8 folds.

**Conclusion:**

vB_KquU_φKuK6 cannot be directly used for phage therapy because it is a temperate bacteriophage. However, genetically modified strains of vB_KquU_φKuK6 alone or combined with antibiotics or other lytic *Klebsiella* phages can have therapeutic utilities in treating *K. quasipneumoniae* infections.

## 1 Introduction

Antibiotics are the first-line therapeutic agents in treating bacterial infections, but pathogenic antibiotic-resistant bacteria (ARB) have become a formidable challenge to this norm. Despite the superior healthcare infrastructure in the United States, the Center for Disease Control and Prevention (CDC) estimates that about 2.8 million US citizens (about 1% of the population) contract ARB infections, and about 35,000 die of such infections every year (CDC, 2019). The World Health Organization (WHO) estimates that ARB infections kill over 0.7 million patients per year globally, and the number would rise to 10 million per year by 2050 if new therapeutic interventions to combat ARB infections are not found (Mancuso et al., 2021). The severity of the crisis led the WHO to declare that a “post-antibiotic” era, where common bacterial infections can be fatal, is imminent (Reardon, 2014). Experts from the European Center for Disease Prevention and Control (ECDC) and the US Center for Disease Control and Prevention (CDC) categorized ARBs as multidrug-resistant (MDR) if the isolates are “non-susceptible to ≥1 agent in ≥3 antimicrobial categories,” extensively drug-resistant (XDR) if the isolates have “non-susceptibility to at least one agent in all but two or fewer antimicrobial categories (i.e., bacterial isolates remain susceptible to only one or two categories),” and pan drug-resistant (PDR) if the isolates show “non-susceptibility to all agents in all antimicrobial categories” (Magiorakos et al., 2011).

*Klebsiella pneumoniae* is among the WHO-designated six highest-priority ARB pathogens besides *Enterococcus faecium, Staphylococcus aureus, Acinetobacter baumannii, Pseudomonas aeruginosa,* and *Enterobacter* sp. (WHO, 2017). In 2015, the species *K. pneumoniae* was divided into three species, *K. pneumoniae, K. quasipneumoniae,* and *K.* var*iicola,* based on the genomic diversity of the species (Holt et al., 2015). About 2–4% of clinical infections attributed to *K. pneumoniae* are caused by *K. quasipneumoniae* (Chew et al., 2021; Long et al., 2017). *K. quasipneumoniae* strains constitute up to one-third of the hospital bacterial isolates in some Asian countries (Chew et al., 2021; Crellen et al., 2019). Disease signs due to *K. pneumoniae* or *K. quasipneumoniae* infections are similar (Chew et al., 2021), and like *K. pneumoniae*, *K. quasipneumoniae* has MDR and hypervirulent strains (Altayb et al., 2023; Arabaghian et al., 2019). Furthermore, *K. quasipneumoniae* strains readily acquire antibiotic-resistance genes and plasmids from other species of Enterobacteriaceae (Mathers et al., 2019). Altogether, *K. quasipneumoniae* is an important emerging bacterial pathogen.

The principal strategies to prevent and combat antibiotic resistance include infection control, containment, judicious use of antibiotics, finding new and more effective antibiotics, regulating clinical and industrial use of antibiotics, public education, and global surveillance (Ventola, 2015). In addition, phage therapy, the application of naturally occurring or genetically manipulated lytic bacteriophages, is suggested as an “alternative to antibiotics” in treating infections caused by MDR and XDR pathogens (Romero-Calle et al., 2019; Lin et al., 2017, reviewed by Strathdee et al., 2023). Although veterinary and clinical phage therapy was initiated by Felix d’Herelle (1873–1949), one of the discoverers of bacteriophages, in 1921 (Keen, 2012), phage therapy is not yet approved as a routine therapeutic method by the US Food and Drug Administration and European Medicines Agency, indicating some uncertainties regarding availability, quality assurance, efficacy, safety, and public trust in phage therapy. However, case-by-case compassionate applications and clinical trials of phage therapy have been approved (Yang et al., 2023).

Research in phage therapy gained momentum in 2021 when public funding for research on phage therapy was initiated in the United States, followed by Australia, the UK, and some other member nations of the European Union (National Institute of Allergy and Infectious Diseases, 2024; New South Wales Government, 2024; Fabijan et al., 2023). Worldwide, about 90 clinical trials of phage therapy including four in the United States are currently being carried out with more in the pipeline (Balthazar, 2024). The present study screened, identified, and characterized a strain of novel bacteriophage that efficiently kills a clinical isolate of *K. quasipneumoniae*. This study labels a few challenges in finding new bacteriophages for phage therapy and suggests some amends.

## 2 Materials and methods

### 2.1 Bacterial strains

*Klebsiella quasipneumoniae* strain K6 700603 (previously known as *Klebsiella pneumoniae* Strain K6) was obtained from the American Type Culture Collection (Manassas VA). Additional strains of *Klebsiella pneumoniae, K. oxytoca*, *Escherichia coli, Pseudomonas aeruginosa, Citrobacter freundii*, *Proteus mirabilis, Salmonella enterica,* and *Serratia marcescens* were obtained from ATCC or BEI Resource Depository (Manassas VA).

### 2.2 Bacterial culture, phage screening, and phage titration

Bacterial strains (including *K. quasipneumoniae*) were grown and infected by bacteriophages in trypticase soy broth (TSB) containing 0.2% maltose and 10 mM MgSO_4_, or TSB containing 1.5% agar (TSA plates). The overlay semi-solid medium contained 0.6% agar. Bacteriophages were sampled from wastewater processing facilities in the Utah Valley area. Raw wastewater (10 mL) was transported to the laboratory on ice, centrifuged at 13,000 × g for 3 min to remove cells and particulates, and passed through 0.2-micron Acrodisc Supor^™^ membrane low protein-binding filters (Pall Lab, New York, NY) before use. Phages were enriched by mixing 0.5 mL of the filtrate, 0.5 mL of overnight bacterial culture of the host bacterium (*K. quasipneumoniae*), and 9 mL of TSB, incubating the mixture at 37°C overnight with moderate agitation (250 rotation/min). The culture supernatant was processed similarly to wastewater and then serially diluted with TSB for phage titration. For titration, the host bacterium (*K. quasipneumoniae*) growing in the log phase (20 mL, A600 = 0.4–0.5) was harvested by centrifugation at 5,000 × g for 5 min, and the pellet was suspended in 20 mL of cold 10 mM MgSO_4_ solution. For plaque assays (done in duplicates), 0.1 mL of the 10-fold serially diluted phage samples were mixed in a 13 mL tube with 0.1 mL of the bacterial suspension and incubated for 20 min at 37°C. Melted overlay agar medium (3 mL) adjusted at 50°C was added to the tube, mixed on a Vortex Genie (USA Scientific, Ocala FL), and poured on top of prewarmed (to 37°C) 10-cm TSB agar plates. The plates were sealed with parafilm, inverted, and incubated at 37°C for 16–20 h. The number of plaque-forming units (PFU) was counted by placing the plates on a transilluminator using the formula N = n/(dv), where N = PFU/ml of the undiluted phage sample; n = number of plaques on the plate, d = dilution factor, and v is the volume of virus suspension (in ml) used for infection. Plates having 40–400 plaques were counted.

### 2.3 Plaque purification and storage of phages

Viruses were plaque-purified through four rounds of bacterial infection. In each round, a sterile cotton-tipped applicator or a sterile 200-μl pipette tip was used to touch a well-isolated plaque, and the applicator was washed with 1 ml TSB in a microfuge tube. The wash was filtered, 10-fold serially diluted, and then used for infecting bacteria as described in the previous section. In the fifth round, the phage was mass-produced by infecting 50 mL of host bacterial culture. For short-term storage, the phage-infected bacterial culture was refrigerated. A previously described method (Clark, 1962) was utilized for the long-term storage of plaque-purified phages. Briefly, the culture medium was centrifuged (at 13,000 × g), filtered using a 0.45-micron Acrodisc Supor^™^ membrane low protein-binding filter (Pall Lab), and then an equal volume of sterile glycerol was mixed with the filtrate, the mixture was chilled in a refrigerator and then stored at −80°C.

### 2.4 Phage precipitation and DNA extraction

Phage particles were precipitated for DNA extraction as described previously (Antibody Design Laboratories, 2024). Briefly, the phage-infected bacterial culture was centrifuged at 13,000 × g for 5 min, and the supernatant was filtered using a 0.45-micron filter cartridge. To 12 mL of the filtrate, 3 mL of 5× PEG-NaCl solution (20% PEG8000, 2.5 M NaCl) was added and mixed by inversion. The mixture was incubated on ice for 1 h and then centrifuged at 13,000 × g for 3 min to precipitate the phage particles. The pellet was suspended in 1 ml of 1× Tris-buffered saline (50 mM Tris–HCl pH 7.5, 0.15 M NaCl). The suspension was incubated on ice for 1 h. The suspension was centrifuged at 13,000 × g for 1 min. The clear phage solution was transferred to a new tube.

Before DNA extraction, the bacteriophage preparation (in 0.5 mL of Tris-buffered saline, pH 7.5) was treated with 20 units/mL of DNase I (NEB, Beverly MA) and 0.02 μg/mL of RNaseA (Roche, Indianapolis IN) at 37°C for 1 h, to remove any nucleic acids of the host bacterium. DNase was then inactivated by heating the sample to 75°C for 5 min, and the sample was treated with 1 mg/mL of proteinase K (ThermoFisher, Waltham MA) in a buffer containing 0.1% SDS at 55°C for 1 h. The sample was then cooled to room temperature and extracted with an equal volume of phenol-chloroform-isoamyl alcohol reagent (ThermoFisher) on a rocking platform for 1 h. The mixture was then centrifuged for 5 min at 13,000 × g and the upper aqueous layer was transferred to a new tube. DNA was ethanol-precipitated following standard protocols. DNA was further cleaned using QIAquick cartridges (Qiagen, Germantown MD). DNA concentration was measured using a NanoDrop spectrometer (ThermoFisher).

### 2.5 DNA sequencing, assembly, and annotation

Phage DNA was sequenced using the MinION flow cell v10.4.1, using the Rapid DNA sequencing kit (Oxford Nanopore, New York, NY). The sequencing run was programmed for 20 h using the MinKNOW software package (Oxford Nanopore). The FASTQ files were assembled into contigs using FLYE (Kolmogorov et al., 2019). The median sequencing coverage was 360x (range 19–1,750×). The assembled genome was annotated with Pharokka v1.2.0.^1^ Nucleotide BLAST^2^ was utilized for comparative viral genomics and to check the presence of any integrated bacterial DNA and anti-CRISPR genes in the bacteriophage genome.

### 2.6 One-step growth curve

The latency period and burst size of the bacteriophage were determined from the one-step growth curve as described (Oliveira et al., 2020). Briefly, bacteria growing in log phage (A600 = 0.4–0.5) were harvested by centrifugation. The cells were suspended in 10 mM MgSO_4_, and the cell count/ml was estimated using the formula: 0.5 MacFarland Standard = 1.5 × 10^8^ colony-forming units (CFU)/ml (McFarland, 1907). The cell volume was adjusted with TSB to 2 × 10^10^ CFU/ml. To accomplish a multiplicity of infection (MOI) of 0.01, 1 ml of the bacterial suspension was mixed with 0.1 mL phage suspension containing 2 × 0^8^ PFU. The mixture was incubated at 37°C for 5 min and then centrifuged at 13,000 × g for 5 min to remove any unabsorbed phages. The cell pellet was suspended in 10 mL of TSB and incubated at 37°C with mild agitation. One ml of post-infection sample was harvested at 10-min intervals over 70 min. The sample was centrifuged at 13,000 × g for 5 min to remove bacterial cells. The supernatant was 10-fold serially diluted, and 0.1 mL of the diluted samples were used to infect bacterial cells using the agar overlay method. The plates were incubated at 37°C for 16–20 h and the phage titer was calculated as described in the previous section. The latency period was determined from two independent experiments from the time point before lysis of the host cells, and the burst size was calculated by quantifying the infection centers (i.e., dividing the average PFU/ml of three higher viral titers by the average of three lower virus titer) (Rivera et al., 2022).

### 2.7 Electron microscopy

Electron microscopy was done at the Brigham Young University Electron Microcopy Facility, Provo UT. Phage particles were harvested from infected host cells in broth culture as described in the previous section, or from webbed overlay culture plates by washing with TSB containing 1.0 mM CaCl_2_. The wash was centrifuged at 13,000 × g for 5 min to remove cellular debris. A 5 μL sample of the clean supernatant/wash was placed on copper grids. The grids were incubated at room temperature for 2 min. The fluid was then removed by touching the margin of the grids with a blotting paper and the grids were stained for 2 min with 0.5% aqueous uranyl acetate solution. The grids were examined, and the images were digitally documented using a Helios NanoLab^™^ 600 DualBeam scope (Hillsboro OR).

### 2.8 Host range and stability of phage particles

The host range of the phage was tested using the spotting method (Huang et al., 2018), by placing 10.0 μl samples of cell-free phages (10^9^ PFU/ml) on a bacterial lawn created using the agar overlay method. The inoculated plates were incubated at 37°C for 20 h before visual examination for plaque formation.

The temperature and pH stability of the phage were tested as described (Oliveira et al., 2020; Khawaja et al., 2016), in the range that can be encountered during storage, transportation, formulation, and administration of therapeutic phages. For temperature sensitivity, cell-free bacteriophages (10^8^ PFU/ml) were incubated at the constant temperature of 37°C, 47°C, 57°C, 67°C, or 77°C for 75 min. We also assayed cell-free bacteriophages stored at −80°C and bacteriophages in infected host cell cultures stored at 4°C. For pH sensitivity, 1 ml cell-free bacteriophage (10^9^ PFU) was mixed with 9 mL of TSB previously adjusted to pH 3, 5, 7, 9, or 11 (by adding NaOH or HCl solutions) and incubated at room temperature for 75 min. The treated samples were 10-fold serially diluted using TSB and the phage titer was determined by the agar overlay method.

### 2.9 Lysogenic infection and lysogen immunity tests

Temperate phages, having lytic and lysogenic life cycles, are generally not desirable for phage therapy (Monteiro et al., 2019), because temperate phages may transduce bacterial genes including virulence factor genes and the integrated proviral genome may prevent lytic infections of the lysogens. Whether the *Klebsiella* phage we isolated causes lysogenic infection of the host bacterium was tested using a previously described protocol (Altamirano and Barr, 2021). Briefly, fresh TSA plates were streaked using a sterile applicator or a 200-μl pipette tip that touched the center of an isolated viral plaque. The plates were incubated overnight at 37°C. Several potential lysogen colonies from the plate were grown to pure culture through sequential agar plate streaking experiments. Lysogeny was tested by PCR using DNA extracted from pure cultures of the potential lysogens (as the test item), uninfected *K. pneumoniae* cells (as a negative control), and cell-free bacteriophages (as a positive control) as the template. The primer pairs used were 5′ CGATCGTCAGCCATGCAAAG3′, and 5′GCGAG TCATTCGATGTTGGC3′. The primers were designed based on the nucleotide sequence of the genome of the *Klebsiella* phage using Primer-BLAST software.^3^ Nucleotide BLAST analyses indicated that the primer pair, part of the DNA Primase Gene of the *Klebsiella* phage, is not related to genomic DNA sequences of any known species of *Klebsiella*. The reaction mixture (20 μL) contained 1x Platinum *Taq* II Master Mix (ThermoFisher), 20 picomoles of the primer pairs, and 20 ng of DNA template. The thermocycler was programmed as the following: 94°C for 5 min (one cycle), 94°C for 30 s, 59°C for 30 s and 72°C for 40 s, (37 cycles), and 72°C for 5 min (one cycle). The amplified DNA fragments were resolved in 6% polyacrylamide gels, stained with ethidium bromide (10 μg/ml), and digitally documented. PCR-positive colonies were considered lysogens. Immunity of the lysogens to the phage was tested by infecting the potential lysogens with the bacteriophage using the spot assay method.

### 2.10 Minimum inhibitory multiplicity of Infection (MIM)

MIM of the phage for the host bacterial strain was tested as described (Nikolic et al., 2022). Briefly, the host bacterium grown overnight was pelleted by centrifugation (5,000 × g for 5 min). The pellet was suspended in 0.1 M MgSO_4_ solution to 0.5 McFarland Standard (~1.5 × 10^8^ CFU/ml). This stock was diluted 1:10 with TSB, and then 0.1 mL of the diluted bacterial preparation (~1 × 10^6^ CFU) was seeded into a 96-well plate. The virus preparation was 10-fold serially diluted (10^9^–10^1^ PFU/ml), and 0.1 mL of the diluted virus preparation was added to the wells of the 96-well plate, in triplicates. Uninfected cells (0.1 mL bacterial cells mixed with 0.1 mL of TSB) served as the control. The plate was incubated at 37°C for 20 h. At that point, 20 μl of 0.1% aqueous solution of 2,3,5 triphenyl tetrazolium chloride (TTC) (Sigma-Aldrich, St. Louis MO) was added to the plate. The plate was incubated for another 3 h for any surviving bacteria in the wells to reduce TTC (a colorless chemical) to 1, 3, 5 triphenyl formazan (TPF), a red dye. The MIM is the lowest MOI of phage solution that prevented bacterial growth, indicated by a complete lack of TPF synthesis.

### 2.11 Inhibition of biofilm formation by the bacteriophage

*K. quasipneumoniae* is a biofilm-forming bacterium, and the ability to form a biofilm is a crucial virulence factor of *Klebsiella* spp. (Guerra et al., 2022). Whether the bacteriophage interferes with the bacterium’s capacity to form biofilm was tested as described previously (Bernal-Bayard et al., 2023). Briefly, 1×10^6^ CFU (in 0.1 mL) of bacteria growing in the log phase were seeded into a 96-well plate. Ten-fold serially diluted bacteriophage preparation (0.1 mL, 1 × 10^9^–1 × 10^1^ PFU/well) was also added to the wells in triplicates. Uninfected *Pseudomonas aeruginosa* (ATCC 27853), and uninfected *K. quasipneumoniae* cells served as the controls. The plate was incubated at 37°C for 24 h. Bacterial growth was measured using a BioTek Synergy I plate reader (Agilent, Santa Clara CA) at 600 nm. The liquid medium was aspirated, and the plates were washed thrice with water and then stained for 15 min using 0.2 mL of 0.5% aqueous solution of crystal violet. The crystal violet solution was then rinsed by repeatedly immersing the plate in tap water. The plate was air-dried for 10 min and 0.2 mL of 30% glacial acetic acid solution was added to the wells. After incubating for 5 min at room temperature, 0.18 mL of the fluid was transferred to a new 96-well plate. The absorbance (A585) of the solution was measured using a BioTek Synergy I plate reader.

### 2.12 Phage-antibiotic synergy (PAS)

PAS refers to a decrease in the inhibitory/lethal dose of an antibiotic to a bacterium in the presence of a virus that infects the bacterium (Comeau et al., 2007). Initially, antibiotic sensitivity of the bacterial strain was tested following the standard Kirby-Bauer Disk Diffusion Susceptibility test protocol (Hudzicki, 2009) in 15-cm Muller Hinton Agar plates using antibiotic-impregnated Sensi-Disks^™^ (BD BBL, Franklyn Lake NJ) and the zone of inhibition (ZOI) was measured. The minimum inhibitory concentration (MIC) of the two antibiotics to which the bacterium was found susceptible was then determined. Overnight-grown bacterial culture was centrifuged, and the pellet was suspended in 0.1 M MgSO_4_ to 0.5 McFarland standard. The preparation was further diluted to 1:10 with TSB, and 0.1 mL of bacterial suspension (~1 × 10^6^ CFU) was seeded into a 96-well plate, in triplicates. The selected antibiotics were diluted to 512 μg/ml to 1 μg/ml and 0.1 mL of the diluted antibiotic was added to the wells (i.e., the final antibiotic concentration was 256 to 0.5 μg/ml). The plate was incubated at 37°C for 20 h and bacterial viability was tested by TTC conversion assay as described in the previous section. The lowest concentration of the drug that prevented conversion of TTC to TPF was considered the MIC.

PAS was tested using the checkerboard method as described previously (Nikolic et al., 2022). Briefly, 0.05 mL of a diluted bacterial preparation (~1 × 10^6^ CFU) was seeded into a 96-well plate. Two-fold serially diluted antibiotic solutions (0.05 mL) were added to the wells in rows to the final concentration 1–256 μg/ml. Ten-fold serially diluted bacteriophage preparations (0.1 mL, 10^9^–10^1^ PFU/well, corresponding MOI 1,000 to 0.000001) were added to the wells in columns. The experiments were repeated once. The plate was incubated at 37°C for 20 h. Bacterial viability was tested by reduction of TTC to TPF as described in the previous section. The fractional inhibitory concertation (FIC) was determined by the relation FIC = (MIC_phage-antibiotic combination_/MIC _antibiotic alone_).

## 3 Results

### 3.1 Structure of the virus and its genome

**Figure 1:**
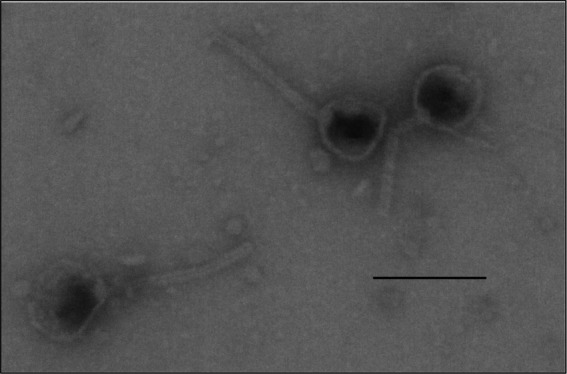
Structure of the capsids of the bacteriophage vB_KquU_φKuK6. Shown is a photograph of electron microscopic images of phage capsids with a roundish icosahedral head (about 60 nm), and a slender tail (about 140 nm × 10 nm), original magnification 349,908×. The bar is 100 nm.

Lytic phages in raw wastewater were screened using *Klebsiella quasipneumoniae* strain K6 (ATCC 700603). A phage isolate that consistently generated large transparent plaques (~2 mm in diameter) was plaque-purified for further analyses. Electron microscopic imaging of the negatively stained samples of the cell-free bacteriophage isolate magnified to 350,000× showed naked complex capsids having an icosahedral spherical head (62 ± 3 nm in diameter) and a slender (137 ± 12 nm × 11 ± 1.36 nm) tail with an inconspicuous tail base plate (Figure 1). At this magnification of the negatively stained samples, the tail fibers, if present, were not visible.

**Figure 2:**
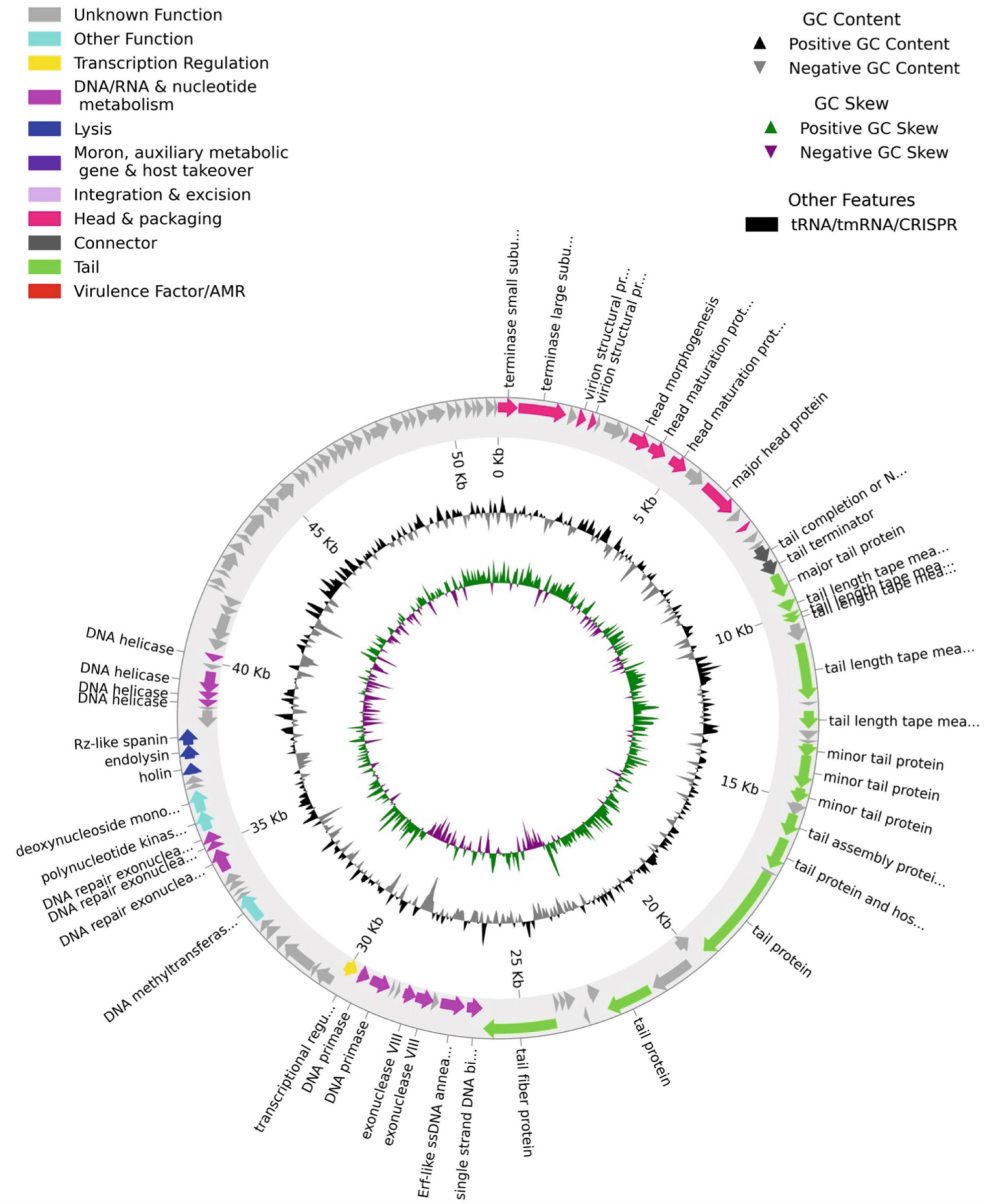
A Pharokka plot for the predicted ORFs of the bacteriophage vB_KquU_φKuK6. The genome is circularized for convenience. Notables: (a) A majority of the ORFs are on the top strand of the genome, (b) ORFs are present both in the AT-rich and GC-rich stretches, (c) about half of the 127 predicted ORFs including all the ORFs in the 3’end of the genome encode proteins of unknown functions, (d) the genome has gene modules for lysogenic infection but not for lysogenic infection.

The nucleotide sequence of the genome of the virus (GenBank accession number is PP874908) indicated that the isolate has an AT-rich (51% AT), linear double-stranded DNA genome 51,251 base pairs in size, containing 121 open reading frames (ORFs). A Pharokka plot of the identified genes and putative genes of the virus are shown in Figure 2. Majority of the genes including all the genes encoding the structural proteins for the head and tail of the capsid and the putative genes are in the top strand of the genome. Genes encoding nucleases, helicases, and enzymes involved in DNA replication (such as primases, and single-strand DNA binding proteins) are in the bottom strand of the genome. The genome contains a putative spanin gene, an endolysin gene, and a holin gene, which are necessary for a lytic infection cycle. The Pharokka v1.2.0. annotation software was unable to detect a genome integration/excision module needed for the lysogenic life cycle. However, the genome contains a gene encoding Erf-like ssDNA binding protein/recombinase in the location 36,705–37,382. A search of the open protein and genome databases using the BLAST software (Altschul et al., 1990) indicated that the genome of the bacteriophage isolate is most closely related to the genome of the *Klebsiella* phage vB_KpnS_SXFY507 (GenBank: ON045001.1) with a Query Cover of 95%, and Percent Identity of 98.17%, and is also related to several other *Klebsiella* phages of the Family *Drexlerviridae*. We named the isolate vB_KquU_φKuK6 (vB- bacterial virus, Kqu- abbreviated for the name of the host species, U- unknown virus genus, φKuK6- the name of the isolate) following the proposed naming convention (Adriaenssens and Brister, 2017).

### 3.2 Host range

Twelve additional strains of gram-negative bacteria (Table 1) were tested to evaluate the host range of vB_KquU_φKuK6. The phage failed to create plaques in the lawn of any of the strains other than *K. quasipneumoniae* within 24 h of the incubation period, indicating its narrow host range.

**Table 1:** Host range of the bacteriophage vB_KquU_φKuK6.

Bacterial strain	Lysis
*Klebsiella quasipneumoniae* Strain K6 (ATCC 700603)	+
*Klebsiella pneumoniae* strain 160_1080 (BEI: NR-44349)	−
*Klebsiella oxytoca* strain MIT 10–5,243 (BEI: HM-624)	−
*Citrobacter freundii* strain ATCC 13316 (ATCC 8090)	−
*Escherichia coli* FDA strain Seattle 1946 (ATCC 25922)	−
*Escherichia coli* stain EDL 931 (ATCC 35150) (BEI: NR-3)	−
*Escherichia coli* stain DH5α (ThermoFisher)	−
*Proteus mirabilis* strain WGLW6 (BEI: HM-753)	−
*Pseudomonas aeruginosa* strain Pa1651 (BEI: NR-51336)	−
*Pseudomonas aeruginosa* strain Boston 41,501 (ATCC 27853)	−
*Salmonella enterica* Tennessee (BEI NR-20742)	−
*Serratia marcescens* Bizio (ATCC 13880)	*−*
*Shigella flexneri* strain serotype 2a (BEI: NR-517)	−
*Shigella sonnei* strain WRAIR I Virulent (BEI: NR-519)	−

### 3.3 One-step growth curve and lytic infection

**Figure 3:**
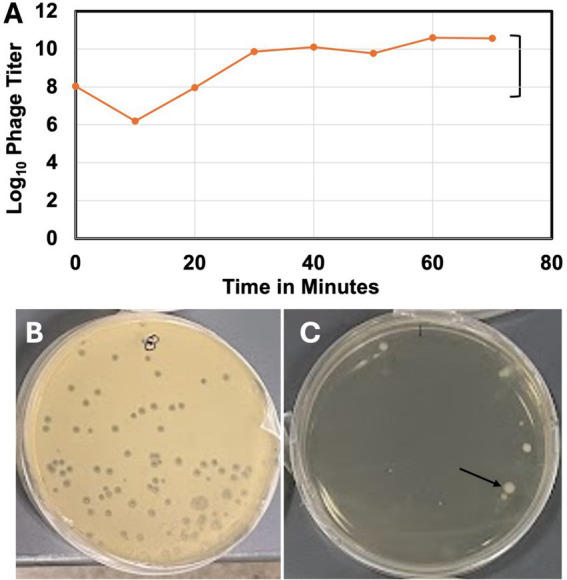
One-step growth curve and some lytic properties of vB_KquU_φKuK6. **(A)** One-step growth curve of the bacteriophage. **(B)** A photograph showing plaques (~2 mm in diameter) created on a bacterial lawn infected at the MOI of 0.001. **(C)** A photograph showing bacterial mesas (indicated by the arrow) on a lawn infected at a MOI of 1,000.

The one-step growth curve of the phage (Figure 3A) indicates a latency period of about 20 min, a growth plateau of about 40 min, and a burst size of 435. Although the phage created large (diameter 2.0 ± 0.13 mm) clear (non-turbid) plaques (Figure 3B), it was unable to lyse the host bacterial lawns to a completion (Figure 3C) or clear the turbid broth culture of the infected host bacterium at any MOI tested (Supplementary Figure 1). At high MOI, bacterial mesas grew in the overlay agar plates (Figure 3C).

### 3.4 Sensitivity to physical and chemical factors

**Figure 4:**
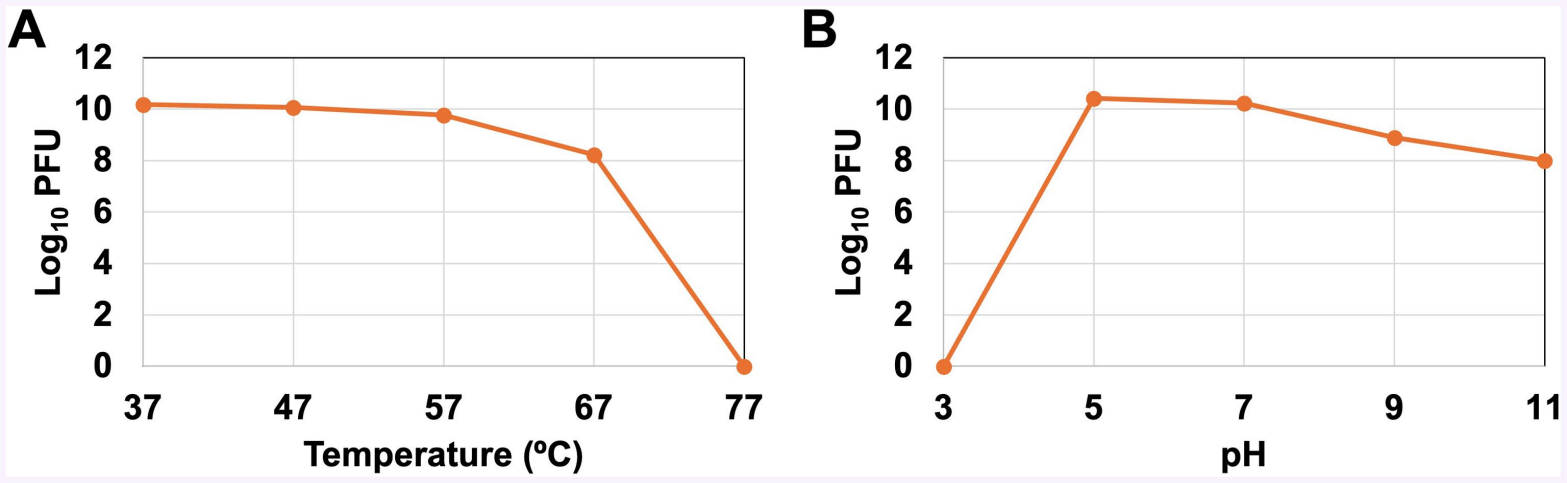
Stability of vB_KquU_φKuK6 at different temperatures and pH. **(A)** Temperature sensitivity. A graph showing log_10_ of phage titer (PFU/ml) after the phage was incubated at indicated temperatures. The phage is unaffected at 37–57°C. Phages incubated at 67°C yielded a titer two orders of magnitude lower. Phages incubated at 77°C were inactivated. **(B)** A graph showing log_10_ of phage titer (PFU/ml) after the phage was incubated at the indicated pH. Phages incubated at pH 3 were inactivated. Phages incubated at pH 5 and pH 7 yielded similar progeny titers. Phages incubated at pH 9 and pH 11 produced a titer reduced by about two orders of magnitude.

The titer of the progeny phages of thermally treated vB_KquU_φKuK6 is shown in Figure 4. There was no reduction in the titer of progeny phages for parental phages treated at 37°C, 47°C, and 57°C. The titer of the progeny phages was reduced by about two orders of magnitude for parental phages treated at 67°C. vB_KquU_φKuK6 treated at 77°C for 75 min was completely inactivated (produced no progeny phage) (Figure 4A). Titer of vB_KquU_φKuK6 refrigerated in infected bacterial culture remained unchanged for at least 4 months and purified vB_KquU_φKuK6 in 50% glycerol stored at −40°C and − 80°C remained biologically active for at least 10 months (Supplementary Figure 2).

The titer of progeny phages of parental vB_KquU_φKuK6 treated at different pH is shown in Figure 4B. Phages treated at pH 3 were inactivated (generated no progeny phage). There was no reduction in the titer of progeny phages for parental phages treated at pH 5 and pH 7. The progeny virus titer was reduced by about two orders of magnitude for parental phages treated at pH 9 or pH 11 (Figure 4B). Phages released by infected host bacterial cells treated with 1% (by volume) of chloroform for 1 min remain biologically active (Supplementary Figure 3).

### 3.5 Lysogenic infection

**Figure 5:**
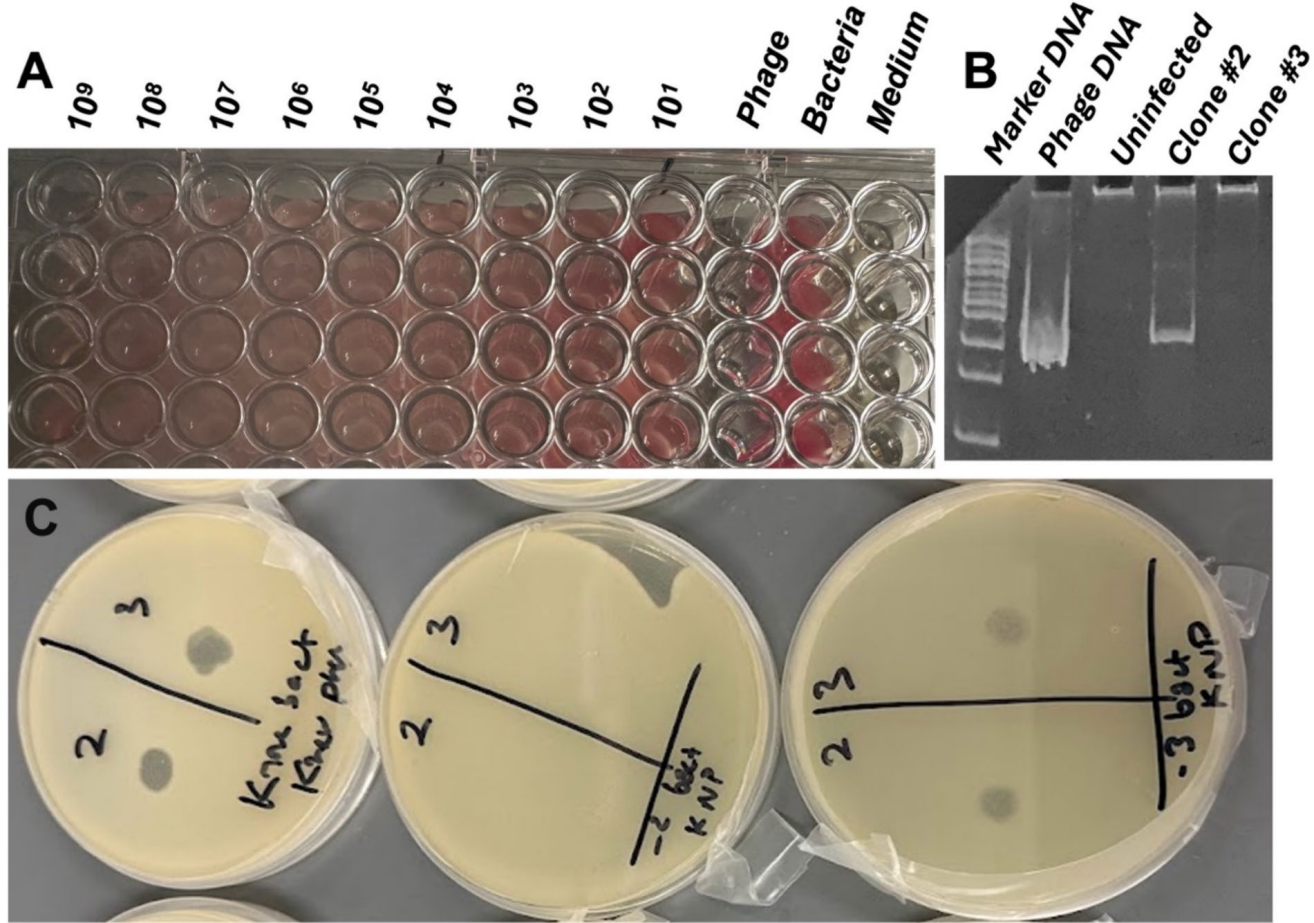
Lysogenic mode of infection by vB_KquU_φKuK6. **(A)** vB_KquU_φKuK6 failed to completely stop the growth of the host bacterium. Shown is a photograph of a part of a 96-well plate growing the host bacterium exposed to different MOIs of vB_KquU_φKuK6 for 20 h. The red dye in the wells indicates the presence of live cells. Phage- phages only, no host bacterium added (the wells were clean; the redness on the right corner of the wells is because of the reflection of the wells on the right side). Bacteria- host cells only, no phage added. Medium- medium only, no bacteria or virus added. **(B)** A PCR-test for lysogenic infection. A photograph of an agarose gel that resolved DNA fragments amplified with a vB_KquU_φKuK6 genome-specific primers. The genomic DNA of plaque-purified vB_KquU_φKuK6 and potential lysogen clone #2 generated the PCR product of the expected size, the genomic DNA of uninfected host cells and potential lysogen clone #3 did not. **(C)** Immunity of the lysogen clone #2 to superinfection. vB_KquU_φKuK6 (10^7^ PFU) was spotted on overlay agar plates seeded with the uninfected host cells (left), clone #2 cells (middle), and clone #3 cells (right). Plaques developed on plates on spots 2 and 3 of the uninfected host cells and potential lysogen clone #3, but the plate growing potential lysogen Clone #2 cells resisted plaque formation.

vB_KquU_φKuK6 reduced growth (measured by A600) of the host bacterium at all MOI (i.e., 0.00001 to 1,000) tested. However, the TTP dye reduction assay indicated that the phage failed to completely halt bacterial growth irrespective of the MOI (Figure 5A), indicating a possible lysogenic mode of infection. To confirm this finding, we isolated potential lysogens from the center of isolated plaques on overlay agar plates to pure culture to examine the genome of the cells for the presence of a potential provirus. PCR tests using vB_KquU_φKuK6 genome-specific primers and the genomic DNA of the isolated potential lysogens as templates indicated the presence of a potential provirus in the genome of the potential lysogen clone #2 (Figure 5B). Spot assay showed that the PCR-positive lysogen clone #2 was resistant to infection by vB_KquU_φKuK6 (Figure 5C), indicating immunity of the lysogens to vB_KquU_φKuK6 reinfection.

### 3.6 Interference to biofilm production by the host bacterium

**Figure 6:**
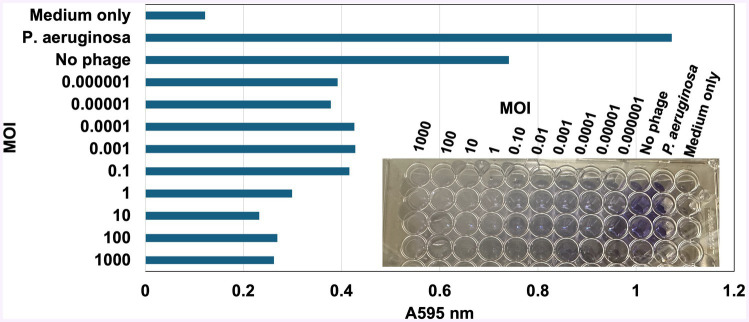
vB_KquU_φKuK6 inhibited the ability of biofilm formation of the host bacterium. Shown is a bar graph of light scattering (A595) by the biofilm formed by uninfected *K. quasipneumoniae* cells (No phage) or the same exposed to a MOI of 1,000 to 0.000001 of vB_KquU_φKuK6. Uninfected *K. quasipneumoniae*, Cells (No phage) uninfected *P. aeruginosa* cells, and uninoculated medium (TSB) served as controls. Inset: A photograph showing biofilm formation indicated by the retention of crystal violet dye in the wells of a repeatedly washed 96-well plate.

vB_KquU_φKuK6 inhibited biofilm production of the host bacterium in a dose-dependent manner, where the higher MOI (1,000-1) reduced biofilm production by approximately 75% and lower MOI (0.1–0.001) reduced biofilm production by about 50% (Figure 6). However, none of the tested MOIs completely negated the ability of the host cells to secrete biofilms.

### 3.7 Phage-antibiotic synergy

**Figure 7:**
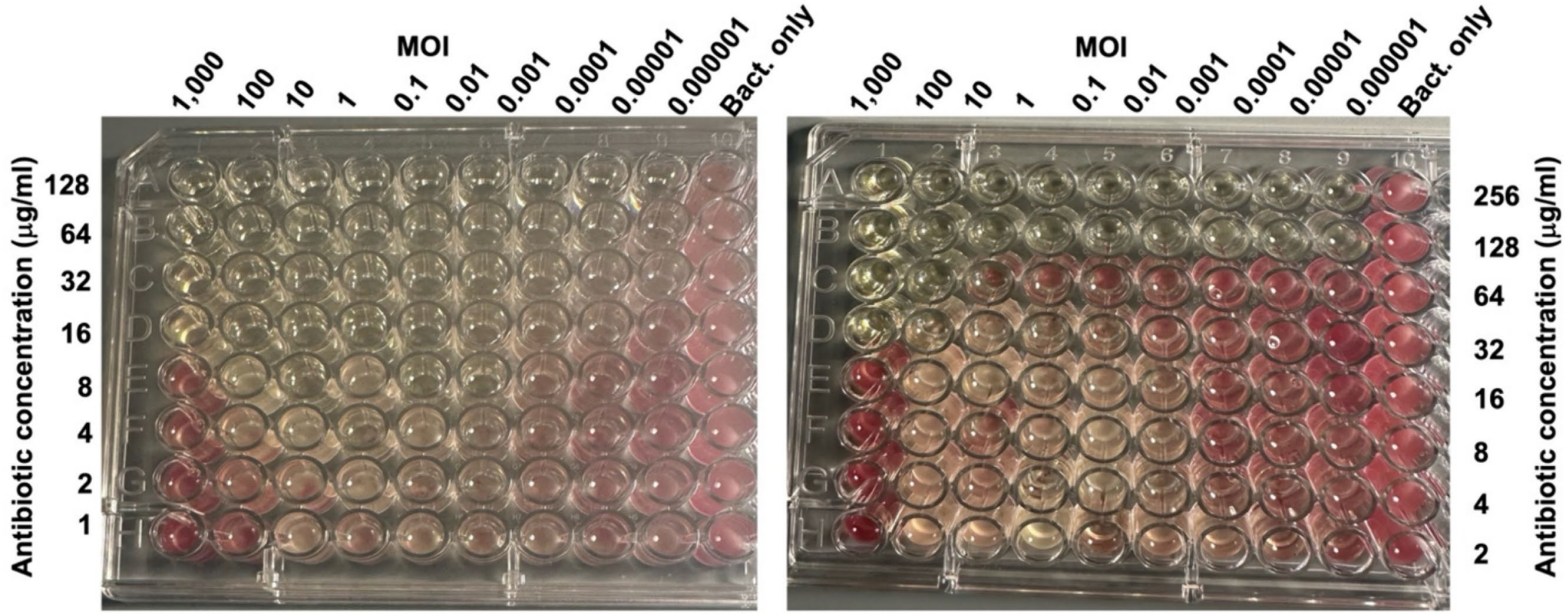
Phage-Antibiotic synergy. Photographs of 96 well plates seeded with the host bacterium (10^6^ CFU) exposed to the indicated MOIs of vB_KquU_φKuK6 (in columns), and the indicated amounts of chloramphenicol (left) or neomycin sulfate in rows (right). The antibiotics or phages were not added to the control wells. The plates were incubated for 20 h and then TTC was added. The picture was taken 3 h after TTC was added. The lack of red color formation indicates complete inhibition of bacterial growth.

Initial disk-diffusion tests indicated that the host bacterial strain is completely resistant (i.e., no ZOI) to cephalothin (30 μg), and erythromycin (15 μg) and resistant/intermediately resistant (Hudzicki, 2009) to chloramphenicol (30 μg, ZOI 11 mm), neomycin sulfate (30 μg, ZOI 8 mm), moxifloxacin (5 μg, ZOI 11 mm), and sulfamethoxazole-trimethoprim (23.75 μg/1.25 μg, ZOI 12 mm) (Supplementary Figure 4). We randomly chose chloramphenicol and neomycin sulfate for further study and determined the MIC for these two drugs to be 64 μg/mL and 256 μg/mL, respectively (Supplementary Figure 5). When phages and antibiotics were combined, bacterial growth was completely inhibited at 8 μg/mL of chloramphenicol in combination with a phage MOI of 0.01, giving a fractional inhibitory concentration (FIC) of 0.125 (Figure 7). However, higher MOIs increased the FIC (for example, the MOI of 1,000 increased the FIC to 0.25). Neomycin sulfate also completely inhibited bacterial growth at 32 μg per ml in combination with a phage MOI of 1,000, giving an FIC of 0.13 (Figure 7).

## 4 Discussion

*K. quasipneumoniae* strain K6 (previously named *K. pneumoniae* stain K6) was isolated from the urine of a hospitalized patient in 1994. The strain has been extensively characterized (Rasheed et al., 2000) and its genome has been sequenced and analyzed (Elliott et al., 2016). Pathologists use the strain as the reference strain for bacterial extended-spectrum beta-lactamase (ESBL) production. Although some strains of *K. quasipneumoniae* colonize the gastrointestinal tract of some healthy subjects (Holt et al., 2015), MDR/hypervirulent strains of *K. quasipneumoniae* have been reported from clinically infected subjects (Altayb et al., 2023; Arabaghian et al., 2019). For having MDR, XDR, PDR, and hypervirulent strains, *Klebsiella* spp. has been a model candidate for phage therapy. Several phages have been reported to have potential use in phage therapy against *K. quasipneumoniae* (Bird et al., 2024; Liu et al., 2024) and *K. pneumoniae* (Fayez et al., 2023; Han et al., 2023; Kondo et al., 2023; Lourenço et al., 2023; Peng et al., 2023; Kim et al., 2022; Nazir et al., 2022; Townsend et al., 2021; Zurabov and Zhilenkov, 2021; reviewed in Herridge et al., 2020) over past few years. The present report describes one such phage vB_KquU_φKuK6, adding to this rapidly growing body of information.

vB_KquU_φKuK6 is a typical coliphage with a spherical icosahedral head (about 60 nm) and a long slender tail (about 140 nm × 10 nm). A nucleotide BLAST search indicated vB_KpnS_SXFY507, a phage that infects *K. pneumoniae* strain SFX506 (Feng et al., 2023), as the closest genetic relative of vB_KquU_φKuK6. The size of the head and tail of vB_KpnS_SXFY507 was reported to be 56 nm and 175 nm, respectively (Feng et al., 2023), which is comparable to that of vB_KquU_φKuK6. The total volume of a vB_KquU_φKuK6 particle is about 1.8×10^5^ cubic nanometers (or about 0.0002 cubic micrometers). We initially screened the phage by filtering wastewater using 0.2-micron, low protein-binding filters. After plaque purification, we used 0.45-micron low protein-binding filters because we observed that the phage titer was reduced from typically about 1.5×10^10^ PFU/ml to about 1.5 × 10^8^ PFU/ml (on average, by about two orders of magnitude) if 0.2-micron filters were used. A previous report indicated that the recovery of different phages is negatively affected by certain types of membranes and smaller pore sizes of the filtration devices (Larsen et al., 2023).

The genome of vB_KquU_φKuK6 is made of a linear dsDNA molecule 51,251 bp in size, comparable to the genome (53,122 bp) of its nearest relative vB_KpnS_SXFY507 (Feng et al., 2023). The structure of the capsids and genomes of vB_KquU_φKuK6 and vB_KpnS_SXFY507 indicate that the two phages are different taxonomic entities. The structures of the virion and the genome indicate that vB_KquU_φKuK6 (along with vB_KpnS_SXFY507 and *Escherichia* phage T1) belongs to the Family *Drexlerviridae*. Several bacteriophages of this family that infect *Klebsiella* spp. have been reported and extensive comparative genomic studies on these viruses have been conducted (Ndiaye et al., 2024; Fayez et al., 2023; Feng et al., 2023; Lourenço et al., 2023; Pertics et al., 2023; Nazir et al., 2022; Martins et al., 2022). The genome of vB_KquU_φKuK6 and many of these related viruses have several gene modules such as: (a) DNA packaging module containing genes for terminases and some other proteins involved in incorporating viral DNA into the capsid, (b) morphogenesis modules containing genes encoding the head and tail proteins of the capsid, (c) genome replication/maintenance modules containing genes involved in nucleotide metabolism, genome replication, genome modification, and recombinases, (d) lysis module containing genes encoding proteins involved in the lysis of the infected host cells such as holins, endolysins, and spanins, (e) modules containing over 50 ORFs encoding proteins of unknown function, and a gene for transcription regulation (Fayez et al., 2023; Feng et al., 2023; Pertics et al., 2023; Nazir et al., 2022; Ku et al., 2021). We found no genes for bacterial virulence, or toxin genes, in the genome of vB_KquU_φKuK6, although we cannot rule out the possibility of those genes given the abundance of ORFs coding proteins of unknown function. A previous study also reported a lack of such genes in the genome of a related virus of the Family *Drexlerviridae* (Fayez et al., 2023). We also found no anti-CRISPR genes in the genome of vB_KquU_φKuK6 although some members of the *Drexlerviridae* family have such genes (Kondo et al., 2023; Kumar et al., 2021). Two other reported phages, EKq1, and KL0, that infect *K. quasipneumoniae*, are not closely related to vB_KquU_φKuK6. The genome of EKq1 is about 48.2 kbp in size and the phage has a putative anti-CRSPR gene (Bird et al., 2024). The genome of KL01 is about 44 kb and the virus is a member of Autophagoviridae (Liu et al., 2024).

The lysis module of the genome of vB_KquU_φKuK6 with the hollin, spanin, and endolysin genes indicates a lytic infection cycle. Hollins accumulate in the plasma membrane and create holes that depolarize the infected cell; endolysins degrade the cell wall polysaccharides; and spanins disrupt the inner and outer membranes (Abeysekera et al., 2022). The genome of many members of the *Drexlerviridae* family contains an Erf-like ssDNA annealing protein/recombinase gene (Ndiaye et al., 2024; Feng et al., 2023; Nazir et al., 2022; Kumar et al., 2021). The genes of the Erf-like ssDNA annealing protein/recombinase family encode proteins that bind ssDNA and supercoiled DNA but not dsDNA (Caldwell et al., 2022; Cheong et al., 2022). Thus, it is unclear if these proteins are involved in conserved site-specific recombination (CSSR) needed for phage genome integration and excision. Our initial analysis of the genome of vB_KquU_φKuK6 indicated an absence of an integration/excision genes module needed for lysogenic infection. However, we observed that vB_KquU_φKuK6 fails to completely lyse bacterial culture irrespective of MOI indicating the possibility of lysogenic infection mode. Our molecular and culture-based assays indicated that vB_KquU_φKuK6 is a temperate phage capable of causing lysogenic infection of *K. quasipneumoniae*. Our PCR test for lysogeny does not confirm whether the lysogens have chromosome-integrated proviruses or episomal genomes of the bacteriophage. Thus, the viral infection can be lysogenic or pseudolysogenic (Hobbs and Abedon, 2016). However, our results indicate that the resulting lysogens are immune to lytic infection by vB_KquU_φKuK6. Other phages capable of lysogenic infection of *Klebsiella* spp. have been reported (Kondo et al., 2023). Lysogeny is naturally selected because it is a part of the survival strategies of bacteriophages (Poteete and Fenton, 1983) and is potentially beneficial to the host bacterium (Howard-Varona et al., 2017). Since lytic/non-lysogenic phages are preferred for phage therapy, candidate phages should be screened by molecular and cellular methods for lysogenic infection. Our limited analysis indicates that comparative genome analysis alone may not rule out the possibility of lysogenic infection by a bacteriophage.

Our preliminary analyses indicate that vB_KquU_φKuK6 has a narrow host range. An ideal therapeutic phage should have a somewhat broader host range (it should be able to infect many pathogenic strains of the target species but not the members of the resident normal biota). However, most of the reported *Klebsiella* phages have a narrow host range because of the diversity of the capsule of the host cells and the substrate-specificity of the depolymerase gene of the phages (Beamud et al., 2023). Because of this and other reasons (including the ability of potential lysogenic infection by many phages, and the ability of the host cells to develop resistance to phage infection), a phage cocktail containing many phage species, is suggested for phage therapy (Beamud et al., 2023; Yoo et al., 2023; Abedon et al., 2021).

A temperate phage, although less desirable, can be useful for phage therapy (Monteiro et al., 2019). Therefore, we investigated if vB_KquU_φKuK6 has some useful attributes of therapeutic phages. An ideal therapeutic phage should infect host cells rapidly and the virions should be stable at a wide range of environmental conditions suitable for formulation, storage, and transport of the phage preparations. We observed that vB_KquU_φKuK6 has a short latency period of about 20 min and a bust size of over 400. The vB_KquU_φKuK6 titer plateaued in 40 min to reach 10^11^ PFU/ml within 2–48 h post-infection. vB_KquU_φKuK6 is resistant to a short exposure to 1% chloroform (an agent used in lysing intact infected bacteria to release phages) and quite stable at −80°C to about 57°C and a pH of 4–11. However, vB_KquU_φKuK6 gets inactivated at pH 3, indicating that its oral application would require manipulation of the stomach pH. Of some *Klebsiella* phages of the Family *Drexlerviridae,* VB_Kpn_ZC2 showed a latency period of 22 min, a burst size of 650 PFU, and stability at −40°C to +50°C and pH 4–9 (Fayez et al., 2023). The *Klebsiella* phage IME268 showed a latency period of 30 min, and a plateau time of 90 min, but the phage is sensitive to chloroform, temperature above 50°C, and pH <4 and > 11 (Nazir et al., 2022). The *Klebsiella* phage vB_KpnS_SXFY507 showed a latency period of 20 min, a burst size of 246, stability at 50°C and pH 4–12, and the phage showed a relatively wider host range (Feng et al., 2023). The *Klebsiella* phage KM18 showed a latency period of 20 min and a burst size of 12 (Ku et al., 2021). Overall, several *Klebsiella* phages of the Family *Drexlerviridae* have some good attributes of a therapeutic phage.

Biofilm secretion is a major virulence factor of *Klebsiella* spp. (Guerra et al., 2022) and most other pathogenic bacteria (Domenico et al., 2017). Infection by a lytic phage prevents capsule secretion by the infected bacterium, reducing the possibility of biofilm formation. vB_KquU_φKuK6 reduced biofilm secretion of its host strain by up to 75% in a MOI dose-dependent manner, but the phage failed to completely stop biofilm production at any MOIs tested. A previous report indicated that the *Klebsiella* phage KM18 (Family *Drexlerviridae*) significantly reduced (but was unable to stop) biofilm production by *Klebsiella michiganensis*, by killing host bacterial cells (Ku et al., 2021).

*K. quasipneumoniae* is resistant to many antibiotics (Altayb et al., 2023). vB_KquU_φKuK6 demonstrated phage-antibiotic synergy (PAS) by reducing the MIC of chloramphenicol from 64 μg/ml to 8 μg/m at a MOI of 0.01. However, at higher MOI (of 1–1,000) a higher dose of the antibiotic was needed to stop bacterial growth, indicating that some host cells may become rapidly lysogenized and the lysogens required a higher dose of antibiotics for lysis. vB_KquU_φKuK6 also reduced the MIC of neomycin sulfate from 256 ug/ml to 32 ug/ml. A previous report indicated a *Klebsiella* phage that reduced MIC of cefepime and tetracycline for two MDR strains of *K. pneumoniae* (Qurat-ul-Ain et al., 2021). PAS has been demonstrated in other pathogenic bacteria including biofilm-producing bacteria such as *Pseudomonas aeruginosa* (Holger et al., 2023; Osman et al., 2023). We anticipate simultaneous application of PAS and a phage cocktail to be clinically more effective in treating MDR/XDR bacterial infections.

## 5 Conclusion

We have isolated a new bacteriophage (vB_KquU_φKuK6) that infects and effectively lyses *K. quasineumoniae*, an MDR pathogen. The phage is stable at clinically relevant ranges of temperatures and pH. The phage rapidly infects the host strain and generates a high virus titer. However, vB_KquU_φKuK6 has a narrow host range, it causes lysogenic infections, and the resulting lysogens are resistant to lytic infection by vB_KquU_φKuK6. Despite these shortcomings, vB_KquU_φKuK6 has potential for therapeutic applications because the phage significantly reduces its host’s ability to produce biofilm and the minimum inhibitory concentration of certain antibiotics. Further research is needed to create a genetically modified vB_KquU_φKuK6 incapable of lysogenic infection. Such an agent, preferably in combination with antibiotics and/or other therapeutic *Klebsiella* phages, can be used in treating infections caused by *K. quasineumoniae* and possibly some other strains of *K. pneumoniae.*

## Data Availability

The genomic dataset presented in this study can be found in online repository. The names of the repository/repositories and accession number(s) can be found below: https://www.ncbi.nlm.nih.gov/genbank/, PP874908.

## Footnotes

^1^ https://bear-apps.bham.ac.uk/applications/2022a/pharokka/1.2.0/

^2^ https://blast.ncbi.nlm.nih.gov/Blast.cgi

^3^ https://www.ncbi.nlm.nih.gov/tools/primer-blast/
