# Supplementary material for: Isolation and preliminary characterization of a novel bacteriophage vB_KquU_φKuK6 that infects the multidrug-resistant pathogen Klebsiella quasipneumoniae
Source: Front Microbiol. 2024 Oct 15;15:1472729. doi: 10.3389/fmicb.2024.1472729 (PMC11524547; doi:10.3389/fmicb.2024.1472729)

Supplementary data

Fig. 1. The bacteriophage vB_KquU_φKuK6 could not clear the turbid broth culture of infected *K. quasipneumoniae* at any MOI tested. A. A photograph showing turbidity of uninfected bacterial culture (tube in the middle) and page-infected bacterial cells (MOI 1,000; tube on the right). The tube on the left contains sterile TSB. The tubes were incubated for 20 hours at 37 °C. B. Photograph of a 96-well plate showing bacteria infected by vB_KquU_φKuK6 at a MOI of 10-0.00001. The culture media remained turbid compared to the sterile medium after 20 hours of incubation at 37 °C. Rows A and H and columns 1, 3, and 12 were empty.

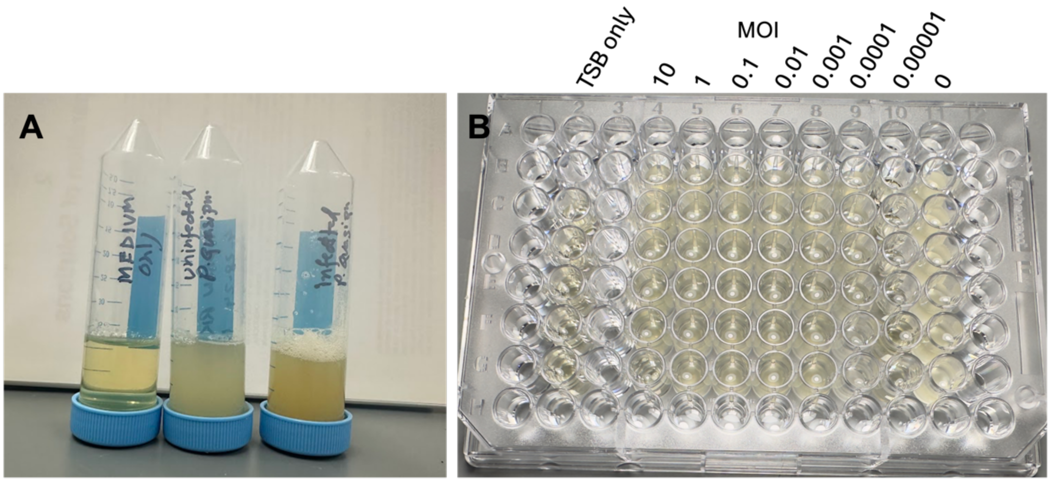

Data Fig. 2. Stability of _KquU_φKuK6 at different storage conditions. A. Bacteriophages titrated after isolating from freshly infected bacterial culture (left), the same infected bacterial culture stored in a refrigerator for three weeks (middle) and three months (right). The titer remained nearly unchanged at about $8x10^8$ pfu/ml. B. Viability of freshly prepared PEG8000-precipitated virus stock (left), PEG8000-precipitated virus stock in 50% glycerol stored initially at -40 °C for two days and -82 °C for five days (middle) and 10 months (right), by spot assay. Dilution factors: 1= undiluted, 2- diluted 1:10, 3- diluted 1:100, 4- diluted 1:1,000, 5-diluted 1:10,000, 6-diluted 1:100,000, 7-diluted 1: 1,000,000, 8- diluted 1: 10,000,000million, 9- diluted 1: 1,000,000,000; a 10 µl aliquot of the samples was spotted on the bacterial lawn. Storage at -82 °C reduced the virus titer by about 2 logs.

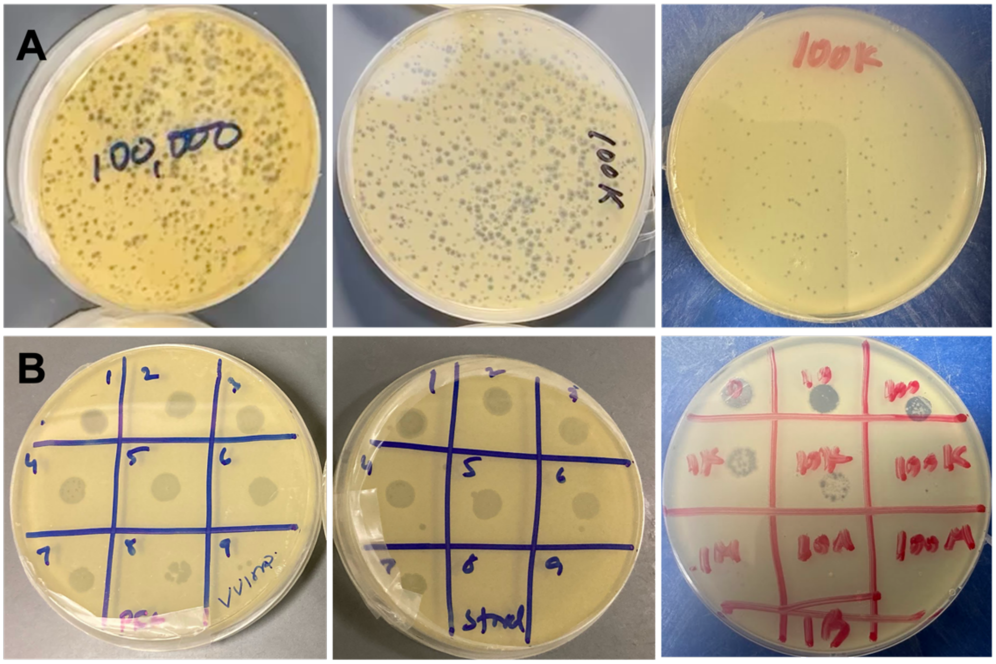

Fig. 3. vB**_KquU_φKuK6 released by chloroform-treated infected *K. qusipneumoniae* remains biologically active.** Phages were collected by filtering one ml of infected bacterial culture medium after mixing with chloroform (1% by volume) for one minute (top) or the same volume of untreated bacterial culture. The phage titer of the filtrate was determined by infecting host bacterial cells using the agar overlay method. The phage titer was about 2-fold higher for chloroform-treated cells (top two plates) compared to nontreated cells (bottom two plates).

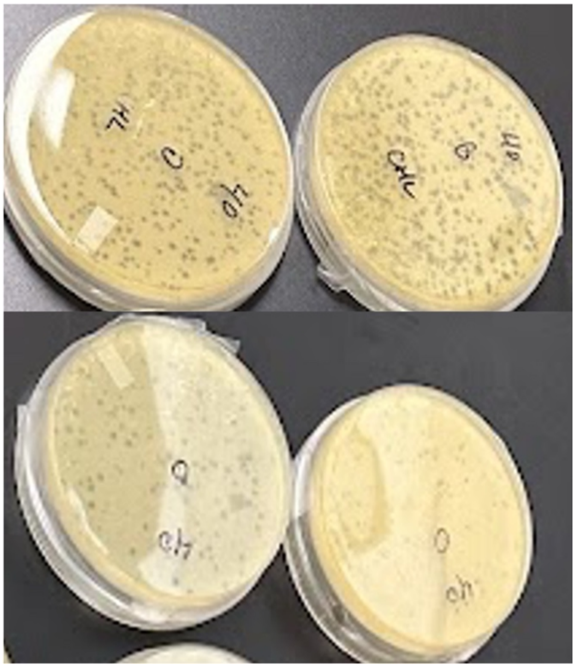

Fig. 4. Antibiotic susceptivity of *K. qusipneumoniae*. A photograph of a Muller-Hinton agar plate seeded with the bacterium showing the outcome of a Kirby-Bauer disc diffusion test. The zone of inhibition indicates that the bacterium is completely resistant to erythromycin (disc #2), penicillin (disc # 5), vancomycin (disc #6), and cephalothin (disc #7); and resistant/intermediate sensitive to sulfamethoxazole-trimethoprim (disc #1), moxifloxacin (disc #3), neomycin (disc #4), and chloramphenicol (disc #8).

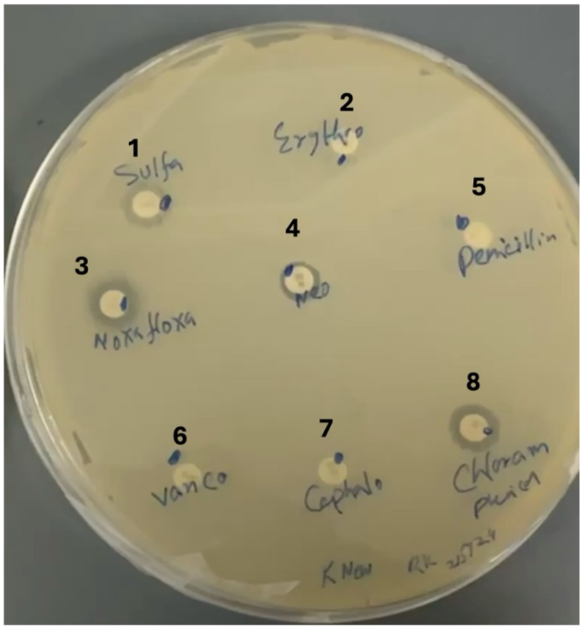

Fig. 5. MIC of chloramphenicol and neomycin sulfate for *K. quasipneumoniae.* A 96-well plate seeded with the bacterium showed the effects of different doses of chloramphenicol (top four rows) and neomycin sulfate (bottom four rows). Wells B1 and E1 were accidentally contaminated, and well C12 received no antibiotics. From two independent experiments, MIC for chloramphenicol was estimated at 64 μg/ml, and that of neomycin sulfate was estimated at 256 μg/ml.

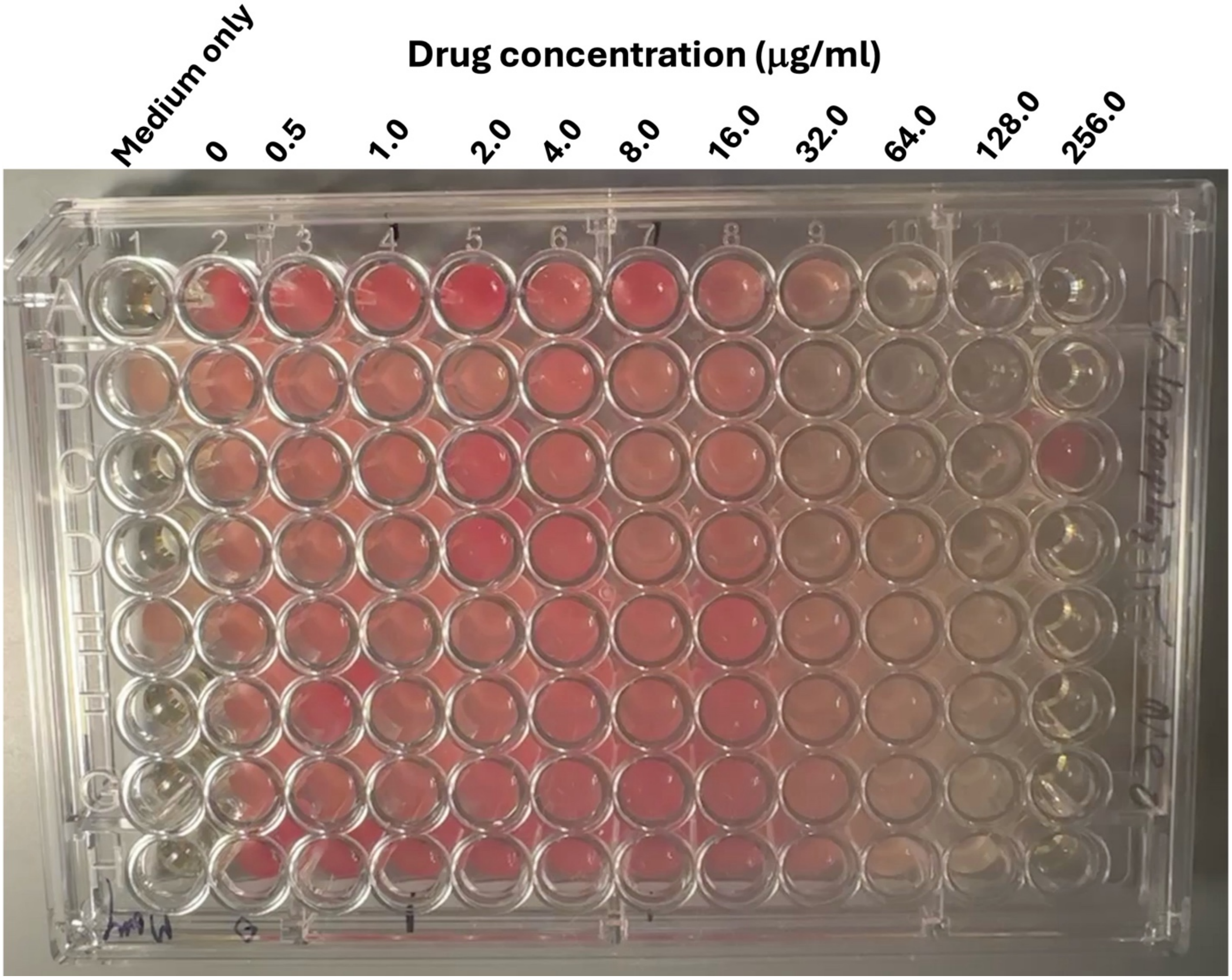

Supplement: Supplementary file 1 [file Presentation_1.pdf]
